# Effect of Maternal Advanced Endometriosis on Risk of Congenital Malformations for Infants Born After *in vitro* Fertilization and Frozen–Thawed Embryo Transfer: Analysis of 28,600 Newborns

**DOI:** 10.3389/fendo.2019.00763

**Published:** 2019-11-12

**Authors:** Zhou Liang, Mingru Yin, Meng Ma, Yun Wang, Yanping Kuang

**Affiliations:** Department of Assisted Reproduction, Shanghai Ninth People's Hospital, School of Medicine, Shanghai Jiao Tong University, Shanghai, China

**Keywords:** endometriosis, IVF, congenital, malformation, frozen–thawed embryo transfer

## Abstract

**Background:** Endometriosis is one of the most challenging diseases for doctors helping infertile women conceive, which has become a common method to help maternal endometriosis-associated infertility. Women with advanced endometriosis possess a higher risk of several adverse outcomes both during pregnancy and at the time of delivery. Whether endometriosis gives rise to a higher occurrence of congenital abnormalities in infants via *in vitro* fertilization and frozen–thawed embryo transfer (IVF-ET) remains unknown.

**Methods:** Data collected on 22,865 women undergoing IVF using a freeze-all strategy from 2007 to 2017 were analyzed to estimate the rate of congenital malformations. We used an adjusted OR to compare the fertility outcomes of women with advanced endometriosis to the control group.

**Results:** We studied 1,495 infants born from women with advanced endometriosis and 27,105 infants born from endometriosis-free women. There was a 1.557-fold risk that the infants with advanced maternal endometriosis would develop a congenital malformation (adjusted OR: 1.557, 95% CI: 1.03–2.35). Compared with singletons, twins were 1.957 times more likely to experience an adverse outcome (OR: 1.957, 95% CI: 1.561–2.455). When analyzing specific categories of birth defects, the proportion of circulatory system defects was higher than the other categories of birth defects in total (0.56%), followed by musculoskeletal system defects (0.15%).

**Conclusions:** Maternal advanced endometriosis might increase the risk of congenital malformations for infants born after IVF-ET. The organ system most frequently affected by congenital malformations was the cardiovascular system, followed by the musculoskeletal system.

## Introduction

The disease in women, endometriosis, is identified by endometrial glands, also known as stroma in sites besides the uterine cavity. Normally, the lesion is located in the ovaries or the peritoneum. Doctors have to face immense challenges while helping infertile women conceive due to endometriosis. It affects over 150 million women worldwide and is also estimated to affect 7–12% of women of reproductive age; there is a much higher incidence in infertile women. In total, 25% of patients undergoing assisted reproductive technology (ART) are affected and 20–40% of these patients show ovarian endometriosis (1, 2).

Several etiologies, such as oxidative stress (3), inflammatory factors, cytokines (4), genetic etiology (5, 6), immunity (7), and hormone role (8), have been reported as catalysts for endometriosis.

Though it is known that the prevalence peaks of the disease occur in the reproductive age, endometriosis leads to complications in both pregnancy and conception. Women with endometriosis have failed to draw the attention on the eminent personalities toward the health of children born to them until recently. Recent studies have revealed that pregnancy complications are more common in women inflicted with endometriosis, leading to miscarriage, and ectopic pregnancy at times (9). Moreover, it has been substantially proven from the start that premature births are more prevalent in women with endometriosis and pose a major health hazard (10–13), despite the existence of conflicting results (14, 15). Women with advanced endometriosis lead to enhanced risks with adverse results in pregnant women and the time of delivery. Besides, advanced endometriosis has given rise to increased risk of neonatal deaths and unfavorable obstetrics in women (16).

Now, one of the most preferred methods to help maternal endometriosis-associated infertility is *in vitro* fertilization (IVF). The natural cycles that are inflicted by the presumably disturbed functions due to endometriosis can be bypassed with the use of IVF and frozen–thawed embryo transfer (IVF-ET). However, there is pretty little knowledge and information regarding the higher incidence of congenital abnormalities in infants through IVF-ET to women with advanced endometriosis.

The purpose of this study is to compare the risk of congenital malformations for infants born after IVF-ET in women with or without endometriosis.

## Materials and Methods

### Population Study and Design

The laparotomy or laparoscopy enabled the diagnosis of patients afflicted with endometriosis, while all were treated surgically. The procedure of endometriosis was conducted based on the guidelines of the revised American Society for Reproductive Medicine (ASRM) classification (17). The data in this study were gathered from the database of ART in the Department of Assisted Reproduction of the Shanghai Ninth People's Hospital, under the aegis of the Jiaotong University School of Medicine, Shanghai, China, for the time period between January 2007 and December 2017. The treatment and/or birth taking place in ART are documented and updated in this database. These details of the births and treatments in ART and the resultant birth through ART are required by the Technical Standard for Human Assisted Reproduction under the Chinese Ministry of Health (CMOH). The parental medical condition (pre-existing and gestational) and the neonatal medical data were also recorded for each participant. The information regarding the obstetric and perinatal outcomes and the hospital medical records were provided by women within 3 months after delivery. This study involved the population that conceived using the benefits of ART, whether live or otherwise, with a gestational age of minimum 23 weeks or with weight on birth of not <500 g.

### Follow-up and Definitions

The data in this study were obtained from an IVF database that included all records for IVF treatments and outcomes in patients who have undertaken IVF treatment in the Shanghai Ninth People's Hospital affiliated to JiaoTong University School of Medicine (a large hospital-based tertiary care reproductive center in Shanghai, China) since 2007. In this study, the clinical outcomes of IVF are presented via a patient-anchored approach with all embryo transfer cycles attached to the patient undergoing IVF treatment.

Endometriosis in the advanced form was diagnosed through laparotomy or laparoscopy and following the new guidelines on classification of endometriosis (17) staged as III–IV. The definition of a live birth was given as a birth that displayed any signs of life irrespective of the duration of gestation, based on the definition provided by the World Health Organization. The 10th Revision (ICD-10) of the International Classification of Diseases specified the definition and codes for the birth defects.

### Statistical Analysis

The SPSS 24.0 software (SPSS, Inc.) was used in the statistical analyses. The data were represented with ±SD where they showed normal distributions or as medians (ranges) when they exhibited abnormal distributions. Qualitative data were displayed in percentages. *t*-tests were used to analyze the variations via continuous parametric data, while the rate comparisons between groups were performed through the χ^2^ test or the exact test by Fisher. Chi-square test was used to compare birth characteristics in births after assisted conception and in the total population (including all births in Shanghai). Fisher exact test was applied to assess the significance of the difference in these groups when the expected number in any of the cells was less than five. Quantification of the risk factors leading to congenital malformations between the groups' binary logistic regression was carried out. The adjusted odds ratio (OR) along with a 95 confidence interval (CI) illustrated the influence of the risk factors on congenital malformation.

## Results

### Pregnancy and Delivery Outcomes

In total, 1,495 live infants were born in the endometriosis (EMs) group and 27,105 infants were born in the control group. The baseline maternal attributes across the two groups are listed in Table 1. More pregnancies were experienced by the women in the control group as compared with the women in the EMs group (*P* < 0.01). Besides, the singleton births proportion was more in the EMs group than in the control group (77.1 vs. 74.4%, *P* < 0.01). Other maternal attributes such as infertility duration, body mass index, gestational age, and type of cycles, were similar across both the groups.

**Table 1:** Maternal background characteristics and neonatal outcomes.

	**EMs(*n* = 1,212)**	**Non-EMs(*n* = 21,653)**
**Maternal age (years)**		
<30	102 (8.4)	6,783 (31.3)
30–34	470 (38.8)	9,785 (45.2)
35–39	467 (38.5)	4,540 (21.0)
≫40	173 (14.3)	545 (2.5)
**Infertility duration**		
<5	901 (74.3)	15,915 (73.5)
5–10	295 (24.3)	5,251 (24.3)
>10	16 (1.3)	487 (2.2)
Median	3 (1.5)	3 (1.5)
**Body mass index**	**20.95** **±** **2.61**	**20.89** **±** **2.58**
**Pregnancies**		
0	810 (66.8)	11,747 (54.2)
1	250 (20.6)	5,216 (24.1)
≫2	152 (12.5)	4,690 (21.7)
**Type of cycle**		
IVF	771 (63.6)	13,906 (64.2)
ICSI	310 (25.6)	5,495 (25.4)
IVF + ICSI	131 (10.8)	2,252 (10.4)
**Live born infants**		
Singletons	935 (77.1)	16,104 (74.4)
Twins	277 (22.8)	11,002 (25.6)
**Gestational age**		
<37 weeks	221 (18.2)	3,280 (18.3)
≫37 weeks	991(81.8)	14,653 (81.7)

*Body mass index (20.95 ± 2.61 20.89 ± 2.58) means the BMI in both group were similar without statistically difference*.

Table 2 presents the complete breakdown of the various organ systems and their detected malformations. Around 1.81% of infants born in the EMs group had defects at birth, while the ratio was 1.10% for the control group infants. Multiple birth defect proportions of about 0.13 and 0.08%, respectively, were observed. Circulatory system defects had a higher incidence of defects at birth (0.56%), followed by musculoskeletal system defects (0.15%), than the total birth defects in other categories, revealed by an analysis of the extent of birth defects in specific categories.

**Table 2:** Types of malformations among live-born infants.

	**EMs(*n* = 1,495)**	**Non-EMs(*n* = 27,105)**	**Adjusted OR (95% CI)**
Any defect	27 (1.81)	298 (1.10)	**1.61 (1.08**–**2.40)**
Multiple defects	2 (0.13)	21 (0.08)	**1.39 (0.31–6.27)**
Nervous system (Q00–Q07)	0	13 (0.05)	NA
Eye, ear, face, and neck (Q10–Q18)	2 (0.13)	21 (0.08)	1.61 (0.36–7.26)
Circulatory system (Q20–Q28)	10 (0.67)	149 (0.55)	2.12 (0.99–4.56)
Respiratory system (Q30–Q34)	2 (0.13)	19 (0.07)	1.78 (0.51–14.23)
Cleft lip and cleft palate (Q35–Q37)	2 (0.13)	9 (0.03)	2.71 (0.52–14.18)
Digestive system (Q38–Q45)	0	22 (0.08)	NA
Genital organs (Q50–Q56)	1 (0.07)	8 (0.03)	1.86 (0.18–19.07)
Urinary system (Q60–Q64)	4 (0.27)	16 (0.06)	3.11 (0.83–11.58)
Musculoskeletal system (Q65–Q79)	4 (0.27)	40 (0.15)	1.38 (0.47–4.06)
Chromosomal anomalies (Q90–Q99)	1 (0.07)	4 (0.01)	5.16 (0.50–53.72)
Metabolic abnormalities E00–E90	2 (0.13)	12 (0.04)	1.86 (0.39–8.84)
Other malformations (Q80–Q89)	1 (0.07)	6 (0.02)	4.35 (0.45–41.83)

*Adjusted OR in any defect [1.61(1.08–2.40)] means there was a 1.61-fold risk that the infants with advanced maternal endometriosis would develop a congenital malformation. Adjusted OR in multiple defects [1.39(0.31–6.27)] means there was a 1.39-fold risk that the infants with advanced maternal endometriosis would develop multiple defects congenital malformation*.

Table 3 presents the proportion of birth defects stratified by singletons. Table 4 presents the proportion of birth defects stratified by twins.

**Table 3:** Proportion for birth defects of singleton infants.

	**EMs(*n* = 1,495)**	**Non-EMs(*n* = 27,105)**
Singleton	935	16,104
Any defect	11 (1.18)	167 (1.04)
Multiple defects	1 (0.11)	13 (0.08)
Nervous system (Q00–Q07)	0	8 (0.05)
Eye, ear, face and neck (Q10–Q18)	1 (0.11)	10 (0.06)
Circulatory system (Q20–Q28)	2 (0.21)	74 (0.46)
Respiratory system (Q30–Q34)	1 (0.11)	13 (0.08)
Cleft lip and cleft palate (Q35–Q37)	1 (0.11)	7 (0.04)
Digestive system (Q38–Q45)	0	13 (0.08)
Genital organs (Q50–Q56)	0	3 (0.02)
Urinary system (Q60–Q64)	2 (0.21)	12 (0.07)
Musculoskeletal system (Q65–Q79)	4 (0.43)	26 (0.16)
Chromosomal anomalies (Q90–Q99)	1 (0.11)	2 (0.01)
Metabolic abnormalities (E00–E90)	0	8 (0.05)
Other malformations (Q80–Q89)	0	4 (0.02)

**Table 4:** Proportion for birth defects of twin infants.

	**EMs(*n* = 1,495)**	**Non-EMs(*n* = 27,105)**
Twins	560	11,001
Any defect	16 (2.86)	131 (1.19)
Multiple defects	1 (0.18)	8 (0.07)
Nervous system (Q00–Q07)	0	5 (0.05)
Eye, ear, face, and neck (Q10–Q18)	1 (0.18)	11 (0.10)
Circulatory system (Q20–Q28)	8 (1.43)	75 (0.68)
Respiratory system (Q30–Q34)	1 (0.18)	6 (0.05)
Cleft lip and cleft palate (Q35–Q37)	1 (0.18)	2 (0.02)
Digestive system (Q38–Q45)	0	9 (0.08)
Genital organs (Q50–Q56)	1 (0.18)	5 (0.05)
Urinary system (Q60–Q64)	2 (0.36)	4 (0.04)
Musculoskeletal system (Q65–Q79)	0	14 (0.13)
Chromosomal anomalies (Q90–Q99)	0	2 (0.02)
Metabolic abnormalities E00–E90	2 (0.36)	4 (0.04)
Other malformations (Q80–Q89)	1 (0.18)	2 (0.02)

**Figure 1:**
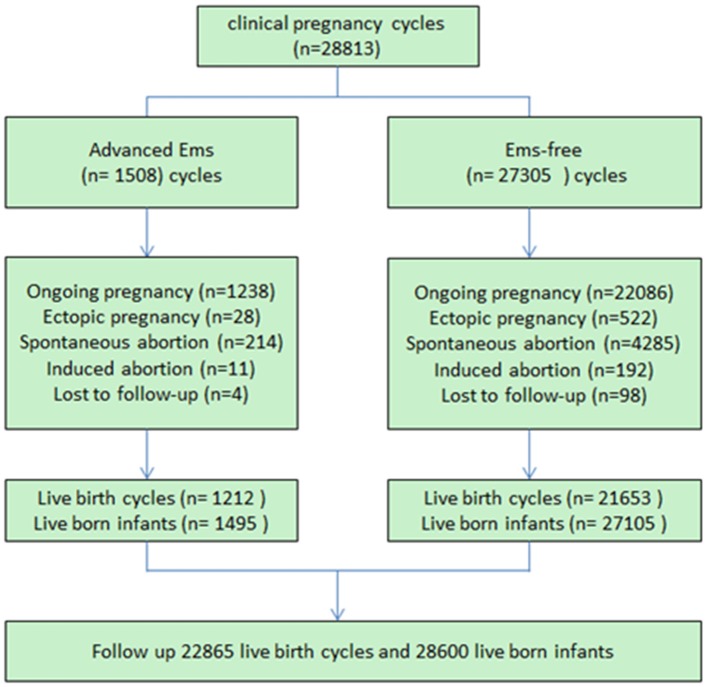
Flow chart of the study.

Logistical analyses exploring the effect of advanced endometriosis on birth defects via IVF treatment, adjusting for maternal age, duration of infertility, pregnancies, BMI, and parity, are listed in Table 5. An increase in the statistical probability of a congenital malformation for EMs and multiple births in the controlled models was illustrated by the results. EMs were found to be associated more likely with increased afflictions of congenital malformation (adjusted OR: 1.557, 95% CI: 1.03–2.35). Figure 1 presents the flowchat of the study.

**Table 5:** Logistic regression for factors influenced congenital malformations.

	**Adjusted OR**	**95% CI**	
**Endometriosis**			
No	Reference	Reference	Reference
Yes	1.557	1.03	2.352
Singleton	Reference	Reference	Reference
Twins	1.957	1.561	2.455
**Years of infertility**			
<5	Reference	Reference	Reference
5–10	0.989	0.752	1.302
>10	1.217	0.678	2.186
**Parity**			
0	Reference	Reference	Reference
≥1	0.848	0.701	1.126
**Age**			
<30	Reference	Reference	Reference
30–34	0.823	0.635	1.067
35–40	0.953	0.692	1.313
>40	0.731	0.378	1.412
**Bmi**			
BMI ≤ 18.4	1.098	0.765	1.577
18.4 < BMI <24	Reference	Reference	Reference
24 ≤ BMI <28	1.290	0.960	1.735
28 ≤ BMI	2.014	1.276	3.179

## Discussion

This study explores the risk of congenital malformations among children born after IVF treatment in women with advanced endometriosis that analyzes different organ systems of the live-born infants.

As some of the confounding variables were different between the two groups, adjusted odds ratios by logistic regression were executed to determine the factors that affected the risk of congenital malformations in offspring. In general, an association among maternal endometriosis and congenital malformations was observed. Multiple pregnancies proved to be a serious risk factor for congenital malformation in consistency with the earlier studies (18, 19).

In women with EMs (22.8%), the occurrence of multiple pregnancies was seen to be lower than that in the control group (25.6%, *P* < 0.01). Moreover, the primiparas in the EMs group were substantially more compared with the control group. These divergences could be explained by the lower implantation rate in the EMs than in the control group. Infertility in endometriosis-afflicted women is known to be influenced by numerous factors such as pelvic adhesions, defective implantation, ovulatory dysfunction, disturbed folliculogenesis (20–25), abnormal expression of proteins, higher FSH levels, and lower anti-Müllerian hormone levels (26). Furthermore, patients with endometriosis revealed an increase in cytokine and inflammatory factor secretion, inducing changes in paracrine-, endocrine-, and inflammation-altered peritoneal environment, distorted pelvic anatomy (27), abnormal uterine transport, and altered hormonal and additional unknown mechanisms (28, 29).

The “junctional zone,” which is another term for the functional and structural abnormalities in the inner myometrium, was observed from the MRI scans and biopsies of women with endometriosis (30–32). The defective transformation of the spiral arteries may be caused by endometriosis, thereby affecting placentation (4). This defective transformation has been documented for contributing to a greater risk of preeclampsia, intrauterine growth restriction, placental abruption, and preterm labor (33). This may also cause the occurrence of birth defects in endometriosis patients.

In our study, the logistic regression showed that the incidence of congenital malformation of twins in both EMs group and the control group were higher than that of singleton pregnancies (adjusted OR = 1.96, 95% CI: 1.56–2.45, *P* < 0.01), which was same as the results of the reference (34, 35). The twins are prone to congenital malformations than the singletons by four times, as has been revealed by certain studies earlier (19). Twinning itself could be the reason for these congenital abnormalities, from vascular connection among the twins or by compression deformation due to uterine crowding. Developmental disruptions are also likely to occur, in the process of twinning, thereby leading to enhanced susceptibility to environmental agents (36). With the increase in the usage of fertility drugs, along with the advancements in assisted reproductive technology, the rate of twinning has also seen an increasing trend, consequently leading to an increased incidence of congenital defects. The factors contributing to the substantially increased risk of birth defects in multiple births have been discussed elaborately in numerous previous studies, which included insufficient nutrition supply, crowded uterine conditions, and common genetic background (18, 37, 38).

In IVF-induced pregnancies, the incidence of birth defects was 1.13% within 7 days of delivery and was found to be in concurrence with the 1.11–1.58% observed in the earlier studies, as reported in a Chinese population-based study (39).

Very few previous studies have discussed the maternal factors' influence on congenital malformation. Only two studies previously focused on the boys having anomalies with their genitals among boys delivered by women having endometriosis (without IVF). Possible linkage with specific factors from the maternal angle and the genital anomaly cryptorchidism (undescended testis) was investigated by a case–control study by Mavrogenis in 2014. The investigators in that study determined that the risk of cryptorchidism among sons born to mothers with endometriosis was double (40). Nevertheless, a register-based Danish study in 2017 revealed the lack of any substantial evidence for the greater incidence of endometriosis-afflicted women giving birth to boys with genital anomalies (41).

Stratifications for organ systems in singleton and twin pregnancies are illustrated in Tables 2–4. The cardiovascular system was found to be the most frequently affected organ system by congenital malformations in our cohort study (0.56%), followed by the musculoskeletal system (0.15%). Among the two, the highest adjusted OR values appear in the chromosomal anomalies (adjusted OR: 5.16, 95% CI: 0.50–3.72; *P* = 0.17), urinary system (adjusted OR: 3.11, 95% CI: 0.83–11.58; *P* = 0.09), and cleft lip/palate (adjusted OR: 2.71, 95% CI: 0.52–14.18; *P* = 0.24). Unfortunately, these three results are not significantly different. This may be due to the fact that the sizes of the positive samples in separated systems are too small.

According to the logistic regression, when the age, duration of infertility, parity, BMI, and number of fetus was controlled, it showed that advanced endometriosis has a 1.557-fold risk that the infants with advanced maternal endometriosis would develop a congenital malformation (adjusted OR: 1.557, 95% CI: 1.03–2.35). Compared with singletons, twins were 1.957 times more likely to experience an adverse outcome (OR: 1.957, 95% CI: 1.561–2.455). Furthermore, we compared patients with different BMIs (18.4 < BMI < 24) and it showed that when BMI exceeds 28, there was a 2.014-fold risk that the infants would develop a congenital malformation. Years of infertility and parity (the number of times a female has given birth) have no significant effect to the outcome of the pregnancy. We have set several groups according to age and ran the logistic regression again. There was no statistically significant difference in the incidence of birth defects between age groups. One explanation was that the infant defects rate in the first age group (<30) of maternal EMs was higher than other groups (5/95), which was accidental.

It is pertinent to note that this study was conducted on more than 20,000 live-born infants treated with IVF, which, to date, is the largest number reported by any study on this topic. However, there are some limitations in the current study: (1) The data lacked the complete details of all birth defects, as the rate of malfunctions of congenital nature was calculated using live newborns and pregnancies that were terminated, rather than all those also associated with stillbirths and miscarriages. Hence, the actual rate of congenital malformation could be higher than what our data imply. (2) Albeit the size of the sample in the control group is relatively big, the analytical power to detect the abnormalities in rare incidence measures was limited, like in the case of congenital malformations by different organ systems. (3) Finally, we failed to take into account some significant variables, like screenings of prenatal fetal abnormalities, environmental exposures, family history of birth defects, parental smoking, and maternal smoking. (4) The weakness of a retrospective study for detection of anomalies, particularly without a standardized dysmorphology assessment.

In conclusion, it can be surmised that advanced maternal endometriosis increases the prevalence of congenital malformations in newborns through inducement of IVF. The cardiovascular system was the organ system that seemed to be most frequently influenced by congenital malformations, followed by the musculoskeletal system. The incidence of congenital malformation of twin pregnancies in both the EMs group and the control group was higher than that of singleton pregnancies. These results may provide evidence for endometriosis etiology and treatment research in the future.

## Data Availability Statement

All datasets generated for this study are included in the article/supplementary material.

## Ethics Statement

This study was approved by the Ethics Committee (Institutional Review Board) of Shanghai Ninth People's Hospital. All procedures performed in studies involving human participants were in accordance with the ethical standards of the institutional and/or national research committee and with the 1964 Helsinki declaration and its later amendments or comparable ethical standards. All participants provided written informed consent.

## Author Contributions

ZL contributed to data collection and drafted the article. MY and MM were responsible for the analysis of data. YW and YK participated in the ultimate interpretation of the study data and in revisions to the article. All authors have read and approved the final manuscript.

### Conflict of Interest

The authors declare that the research was conducted in the absence of any commercial or financial relationships that could be construed as a potential conflict of interest.
